# Iodol

**Published:** 1885-12

**Authors:** 


### SELECTIONS.

### Iodol.

Under this name, Professor Vulpius described to the pharmaceutical section of the recent Congress of German Physicians and Naturalists, a new iodine derivative which is apparently destined to play a very important part in the antiseptics of the immediate future. Iodol, or tetraiod pyrrol. ($C_4$ $I_4$ N. H.) is a brown powder consisting of microscopical crystals sparingly soluble in water, but readily dissolving in alcohol, its solubility in the latter being in direct ratio to the strength of the alcohol. It may be heated to 212° F., (100° C.) without change or decomposition, but any increase of the heat beyond this point causes it to give off dense vapours of iodine. It is odorless or nearly so, and while the slightest addition of water to the alcoholic solution will cause a precipitation of iodol, glycerin may be added in large quantities without producing this result. The method of preparing iodol in commercial quantities, is the subject of a German patent, but the principle is briefly as follows: Iodide of potassium in solution is added to animal oil, causing the separation of pyrrol, and the subsequent precipitation of the tetraiod pyrrol or iodol. The chief advantages of iodol, as an antiseptic are that it has no odor, and that it may be used with great freedom in wounds of all descriptions without fear of producing toxication.—*National Druggist.*

